# National Pension Fund for Nurses

**Published:** 1888-02-04

**Authors:** 


### THE NATIONAL PENSION FUND FOR NURSES.

Good progress has been made with this fund during the past week. The actuary has sent in his tables, which have been considered and returned to enable them to be prepared for the prospectus. All the preliminaries and technicalities have been overcome in connexion with the incorporation of the fund with Parliamentary sanction, and the necessary documents will be signed this week. Mr. John Norbury has contributed £250 to the Bonus Fund, as a mark of his appreciation of the many services rendered to his family by nurses. It is expected that the prospectuses will be ready to be issued before the end of February, and all who wish to get the full benefit of the fund should send in their names to the Acting Secretary, 38, Old Jewry, London, E.C., without delay.

Magnus, a recent writer on colour-sense, has arrived at the conclusion that mankind at first saw only white and black, that red was the first colour to be distinguished, and then the other colours in succession towards the violet end of the spectrum. He thinks that red, black, and white alone were recognised at the time the Vedas were written, and that yellow was perceived in the Homeric period; green, blue, and violet being subsequently discriminated. He suggests that the colour-sense is still imperfect, and that the time may come when the ultra-violet rays will be distinguished by the human eye.

